# Dual Size/Charge‐Switchable Nanocatalytic Medicine for Deep Tumor Therapy

**DOI:** 10.1002/advs.202002816

**Published:** 2021-03-01

**Authors:** Wencheng Wu, Yinying Pu, Jianlin Shi

**Affiliations:** ^1^ State Key Lab of High Performance Ceramics and Superfine Microstructures Shanghai Institute of Ceramics Chinese Academy of Sciences Shanghai 200050 P. R. China; ^2^ Centre of Materials Science and Optoelectronics Engineering University of Chinese Academy of Sciences Beijing 100049 P. R. China; ^3^ Department of Medical Ultrasound Shanghai Tenth People's Hospital Ultrasound Research and Education Institute Tongji University Cancer Center Tongji University School of Medicine Shanghai 200072 P. R. China

**Keywords:** nanocatalytic medicine, size/charge switching, solid tumor therapy

## Abstract

Elevating intratumoral levels of highly toxic reactive oxygen species (ROS) by nanocatalytic medicine for tumor‐specific therapy without using conventional toxic chemodrugs is recently of considerable interest, which, however, still suffers from less satisfactory therapeutic efficacy due to the relatively poor accumulation at the tumor site and largely blocked intratumoral infiltration of nanomedicines. Herein, an ultrasound (US)‐triggered dual size/charge‐switchable nanocatalytic medicine, designated as Cu‐LDH/HMME@Lips, is constructed for deep solid tumor therapy via catalytic ROS generations. The negatively charged liposome outer‐layer of the nanomedicine enables much‐prolonged blood circulation for significantly enhanced tumoral accumulation, while the positively charged Fenton‐like catalyst Cu‐LDH released from the liposome under the US stimulation demonstrates much enhanced intratumoral penetration via transcytosis. In the meantime, the co‐released sonosensitizer hematoporphyrin monomethyl ether (HMME) catalyze the singlet oxygen (^1^O_2_) generation upon the US irradiation, and deep‐tumoral infiltrated Cu‐LDH catalyzes the H_2_O_2_ decomposition to produce highly toxic hydroxyl radical (·OH) specifically within the mildly acidic tumor microenvironment (TME). The efficient intratumoral accumulation and penetration via the dual size/charge switching mechanism, and the ROS generations by both sonosensitization and Fenton‐like reactions, ensures the high therapeutic efficacy for the deep tumor therapy by the nanocatalytic medicine.

## Graphical abstract

This work provides a novel ultrasound (US)‐triggered dual size/charge‐switchable nanocatalytic medicine for deep solid tumor therapy by reactive oxygen species generation in responses to both the US and tumor microenvironment (TME), which is believed to provide a feasible and promising strategy for catalytic tumor‐specific therapy.

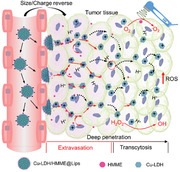

## 1 Introduction

Reactive oxygen species (ROS) are a family of molecules formed by the incomplete reduction of oxygen, including peroxide (H_2_O_2_), singlet oxygen (^1^O_2_), hydroxyl radicals (•OH), among which H_2_O_2_ is the least reactive ROS.^[^ ^1^ ^]^ At adequate concentrations, ROS serve as important second signal messengers both in normal and cancerous cells under the strict regulation of cellular redox balance.^[^ ^2^ ^]^ As a double‐edged sword, the overproduction of ROS will result in serious damages including the oxidization of proteins and damage of DNA structure, thus inducing apoptosis.^[^ ^3^ ^]^ This biochemical property of ROS provides a practical therapeutic approach to kill cancer cells by disrupting the redox homeostasis.^[^ ^4^ ^]^Compared to H_2_O_2_, which is a mild oxidant and specifically overexpressed in tumor cells, ^1^O_2_ and •OH display indiscriminate reactivity and are highly oxidative to all biological targets. Therefore, taking advantage of nanocatalytic medicine and exogenous stimulation to specifically convert intra‐tumoral H_2_O_2_ and O_2_ into •OH and ^1^O_2_ can effectively and safely kill cancer cells without affecting normal tissues.^[^ ^5^ ^]^ However, nanocatalytic medicine, similar to most nanomedicines, usually suffers from hindered diffusion in the solid tumor by the high interstitial fluid pressure (IFP) and dense extracellular matrix severely. As a result, the largely blocked intratumoral infiltration of nanomedicines leads to unsatisfactory therapeutic efficacy, which remains a great challenge in nanocatalytic therapy.^[^ ^6^ ^]^

Numbers of reports have established that slightly negatively charged nanoparticles have a longer circulation time duration in the bloodstream, thus enhancing their tumor accumulation probabilities.^[^ ^7^ ^]^ However, tumor cells will uptake positively charged nanoparticles more preferentially than negatively charged ones. To tackle this issue, the development of nano‐biotechnology enables us to address the aforementioned predicament by designing diverse smart drug delivery systems (DDS).^[^ ^8^ ^]^ For example, various smart nanomedicines with size‐ or charge‐switchable features have been designed to realize deep tumor therapy.^[^ ^7^, ^9^ ^]^ Negatively charged and size‐switchable nanomedicines may not only inherit the long circulatory half‐life in vivo of relatively large (100–200 nm) nanoparticles but is also able to penetrate deep in tumors like relatively small nanoparticles(5–50 nm).^[^ ^8^, ^10^ ^]^ However, the following cell internalization of only size‐switchable nanomedicines is still impeded by their negative surface charges compared to charge‐reversible nanoparticles.^[^ ^11^ ^]^ It has been demonstrated that suitably small size and positive charge favor the infiltration of nanoparticles within tumor tissues through active transcytosis.^[^ ^12^ ^]^ Therefore, designing novel dual size/charge‐switchable nanocatalytic medicine is of great interest for improved therapeutic outcomes in solid tumors.

**Scheme 1:**
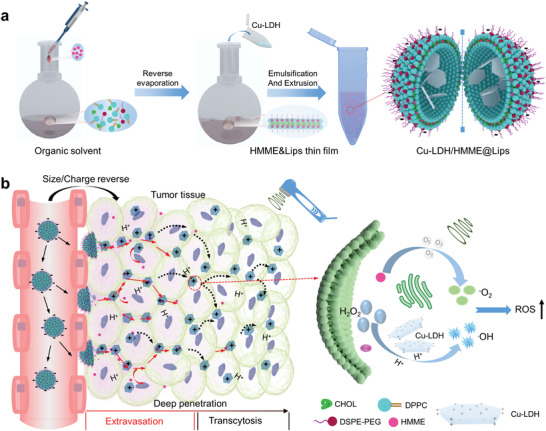
a) Schematic illustration of the synthesis of Cu‐LDH/HMME@Lips. b) Schematic illustration of dual size/charge‐switchable Cu‐LDH/HMME@Lips to transport in poorly permeable solid tumor models and the mechanism of ROS (both ^1^O_2_ and •OH) generations.

Recently, diverse metal‐doped layered double hydroxide (i.e., Fe, Mn) nanosheets with great peroxidase‐like activity have been developed as highly efficient nanocatalytic medicines to trigger the generation of ROS.^[^ ^13^ ^]^ It is noted that the Cu‐based system is able to more effectively initiate Fenton‐like reactions in cancer cells specifically to decompose H_2_O_2_ into highly toxic •OH than conventional Fe‐based Fenton agents.^[^ ^14^ ^]^Herein, we report the synthesis of small‐sized, positively charged, and copper‐doped layered double hydroxide (Cu‐LDH) nanosheets as a novel nanocatalytic medicine. The obtained Cu‐LDH nanosheets are capable of actively infiltrating into tumor tissues through transcytosis for deep tumor therapy. To prolong their circulatory half‐life, small and positively charged Cu‐LDH nanosheets (≈50 nm) were encapsulated into the cores of relatively large liposomes which has a long blood circulation time duration (≈200 nm, Cu‐LDH@Lips) (**Scheme** **1**a).^[^ ^15^ ^]^ In the meantime, the positive charges on the surface of Cu‐LDH nanosheets were camouflaged into the negative one of the liposomes temporarily. Thus, the negatively charged surface of liposomes on a whole endows the nanoplatform with diminished interaction with cells and a resultant much‐prolonged circulation time duration in vivo. Moreover, the sonosensitizers (hematoporphyrin monomethyl ether (HMME)) were embedded into the hydrophobic bilayers of Cu‐LDH@Lips, forming a dual size/charge‐switchable ROS generator named as Cu‐LDH/HMME@Lips. The negatively charged Cu‐LDH/HMME@Lips will circulate in the bloodstream rather stably and gradually concentrate at the tumor site efficiently. After its accumulation, the liposomes can be rapidly disintegrated upon ultrasound (US) irradiation, achieving size switching from the large to small, and concurrently the surface charge reversion from the negative of liposome to the positive of the Cu‐LDH nanoparticles as well, allowing its deep penetration into the tumor tissue through transcytosis. In the meantime and more importantly, the residual O_2_ in the tumor would be converted into ^1^O_2_ under the co‐catalysis by US and HHME. Additionally, the exposed Cu‐LDH nanosheets by the liposome disruption by US irradiation would effectively initiate Fenton‐like reactions to decompose H_2_O_2_ into highly toxic •OH in the mildly acidic tumor microenvironment (TME). Thus the intratumoral levels of both ^1^O_2_ and •OH could be effectively elevated, favoring the deep solid tumor therapy by Cu‐LDH/HMME@Lips under highly penetrating US stimulation. (Scheme 1b)

## 2 Results and Discussion

**Figure 1:**
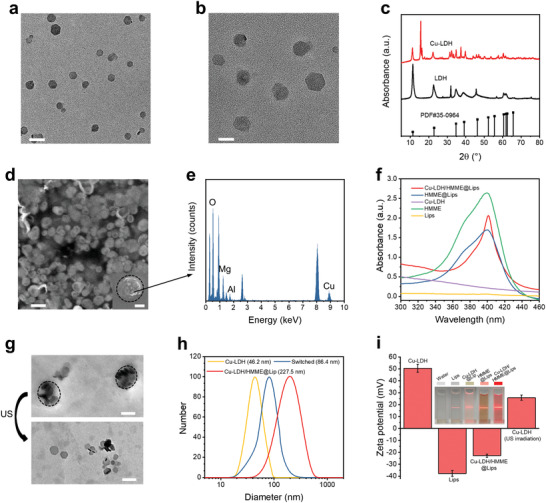
Characterizations of LDH, Cu‐LDH, and Cu‐LDH/HMME@Lips. a) TEM image of LDHs, scale bar, 100 nm. b) TEM image of Cu‐LDH, scale bar, 50 nm. c) XRD spectra of LDHs and Cu‐LDH. d) TEM image of Cu‐LDH/HMME@Lips, scale bar, 500 nm. Insert, SEM images of Cu‐LDH/HMME@Lips, scale bar 100 nm, and e) corresponding energy‐dispersive X‐ray spectrum. f) UV–vis absorbance spectra of Lips, HMME, Cu‐LDH, HMME@Lips, and Cu‐LDH/HMME@Lips, indicating the successful encapsulation of Cu‐LDH and HMME into the liposomes. g) TEM images of Cu‐LDH/HMME@Lips before (up) and after (down) US irradiation. scale bar, 200 nm. h) Hydrodynamic diameters of Cu‐LDH and Cu‐LDH/HMME@Lips before and after US irradiation in PBS measured by DLS. i) Zeta potentials of Cu‐LDH, Lips, Cu‐LDH/HMME@Lips, and Cu‐LDH/HMME@Lip after US irradiation and washed with ethanol, error bars are based on SD (*n* = 3). Insert: digital photos of different corresponding solutions showing distinct Tyndall effects.

To establish the Cu‐LDH/HMME@Lips nanomedicine, Cu‐LDH nanosheets were first synthesized via a two‐step approach. Mg_3_Al‐LDH nanosheets (LDHs) were obtained in advance as a precursor by co‐precipitation, and then isomorphic substitution of partial Mg^2+^ by Cu^2+^ ions was conducted to finally obtain Cu‐LDH nanosheets. Subsequently, Cu‐LDH nanosheets were encapsulated into the hydrophilic cores and sonosensitiser HMME were embedded into the hydrophobic bilayers of liposomes to construct Cu‐LDH/HMME@Lips (Scheme 1a). The prepared LDHs display a plate‐like morphology with a planar size of about 50 nm (**Figure** **1**a), and their morphology and sizes do not change significantly after doping Cu^2+^ (Figure 1b). The chemical composition of Cu‐LDH nanosheets was analyzed by X‐ray photoelectron spectroscopy (XPS, Figure S1a, Supporting Information), in which Cu, Mg, Al, and O elements were detected demonstrating the successful doping of Cu ions. Moreover, two strong characteristic Cu^2+^ satellite peaks at 945 and 962.5 eV were detected in the peak fitting analysis spectrum of Cu 2p, which proves that the oxidation state of Cu ions is Cu^2+^ (Figure S1b, Supporting Information). To further confirm the doping rate of Cu ions, inductively coupled plasma mass spectrometry (ICP‐OES) was used. The molar ratio of Cu to Al in the Cu‐LDH is ≈2:1, and the mass proportion of Cu in Cu‐LDH is ≈26.64% (Table S1, Supporting Information). Furthermore, the XRD patterns of both as‐synthesized LDH and Cu‐LDH display the characteristic LDHs diffraction peaks, and Cu‐LDH exists an obvious characteristic peak at 15° after isomorphic substitution of Mg^2+^ with Cu^2+^ (Figure 1c). After encapsulation into liposomes, Cu‐LDH/HMME@Lips presents a quasi‐spherical morphology and an average diameter of around 200 nm by transmission electron microscopy (TEM) and scanning electron microscopy (SEM) observations (Figure 1d), and the main metal components of Mg, Al, and Cu were detected by EDS analysis (Figure 1e). Additionally, in the UV–vis absorption spectrum of Cu‐LDH/HMME@Lips, the typical HMME peak at 398 nm could be detected, evidencing that HMME had been encapsulated into nanoparticles (Figure 1f).

Next, we explored whether Cu‐LDH/HMME@Lips could achieve the size/charge switching in vitro. It can be found in Figure 1g that Cu‐LDH encapsulated in Cu‐LDH/HMME@Lips are clustered together and presents a quasi‐spherical morphology and relatively large particle sizes before US irradiation. While after US irradiation, Cu‐LDH will be dispersed in solutions and exhibits plate‐like morphology and much‐reduced size, indicating the US‐triggered rupture of Cu‐LDH/HMME@Lips and the release of encapsulated Cu‐LDH nanosheets, i.e., the size switching from the large of the liposome‐based nanoplatform to the small of Cu‐LDH nanosheets. Moreover, the hydrated particle size change before and after ultrasonic treatment determined by dynamic light scattering (DLS) technique also validated the US‐triggered size switching (Figure 1h). According to the results of Zeta potential analysis, the original Cu‐LDH/HMME@Lips are negatively charged before US irradiation, while the Cu‐LDH nanosheets released from Cu‐LDH/HMME@Lips upon US irradiation are positively charged (Figure 1i), indicating the concurrent charge‐switching of the nanoplatforms by this US‐triggered disassembly strategy.

**Figure 2:**
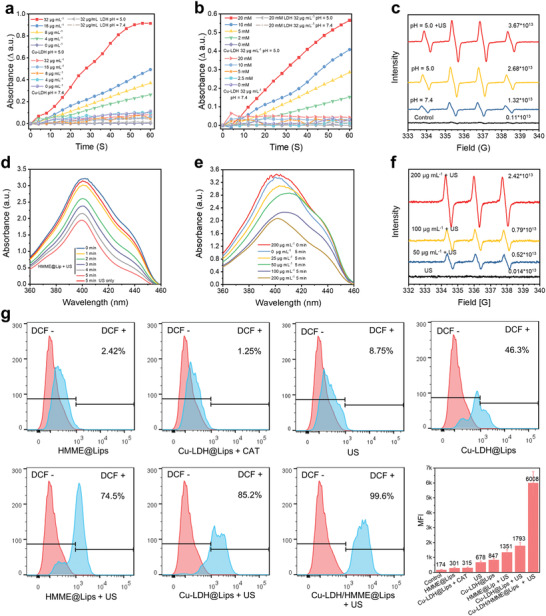
Relative activities of Cu‐LDH in catalyzing H_2_O_2_ decomposition at varied a) Cu‐LDH concentrations and pH values, b) varied H_2_O_2_ concentrations and pH values, determined by measuring the absorbances of the systems at 650 nm via TMB assay. c) ESR spectra of Cu‐LDH@Lips with the addition of H_2_O_2_ in the media of varied pH values by different treatments. d) DPBF absorption spectra by adding 200 µg mL^−1^ of HMME@Lips after US irradiation for varied durations. e) DPBF absorption spectra by addingHMME@Lips of varied concentrations after US irradiation for 5 min. f) ESR spectra of HMME@Lips under US irradiation in media of varied concentrations. g) Flow cytometry analyses and corresponding mean fluorescence intensities (MFI) of ROS generation in 4T1 cells stained with DCFH‐DA after treated in different conditions.

To investigate the peroxidase‐mimicking ability of Cu‐LDH to generate •OH by decomposing H_2_O_2_, the typical colorimetric assay was performed, in which the colorless 3,3’,5,5’‐tetramethylbenzidine (TMB) could be oxidized by •OH species to chromogenic TMB presenting characteristic absorption at 650 nm. As a result, the free Cu‐LDH exhibit catalyst concentration‐, pH‐, and substrate H_2_O_2_ concentration‐dependent catalytic activity (**Figure** **2**a,b). It is worth noting that the catalytic activity of Cu‐LDH nanosheets is significantly suppressed under neutral conditions (pH 7.4), so even if these nanosheets had been taken in the normal cells, the oxidative damage induced by the catalyst to normal tissues would be minimized. Impressively, even at relatively high catalyst and substrate H_2_O_2_ concentrations, the catalytic activity of free LDH could be ignored (Figure 2a,b), which indicates that Cu‐LDH possessed catalytic activity only doped with Cu^2+^. Then, the capability of Cu‐LDH to generate •OH after being encapsulated in liposomes was monitored by electron spin resonance (ESR) spectroscopy, in which 5,5‐dimethyl‐1‐pyrroline N‐oxide was applied as a spin trap of •OH. The substrate H_2_O_2_ (100 × 10^−6^ m) was added to Cu‐LDH@Lips suspensions of different pH values (pH 7.4, 5.0) to simulate the neutral reaction system in normal tissues and mildly acidic reaction system in tumor tissues, respectively. In comparison with the control group, the strong characteristic 1:2:2:1 •OH signal is detected in acidic conditions with a spin concentration of 2.68 × 10^13^ spins mg^−1^, while the •OH signal in the neutral reaction system is less significant (Figure 2c). Particularly, in the mildly acidic reaction system, the •OH signal is significantly enhanced after US irradiation owing to the release of Cu‐LDH from Cu‐LDH@Lips exposing more active sites, and its spin concentration is 3.67 × 10^13^ spins mg^−1^ (Figure 2c). Besides, the 3‐diphenylisobenzofuran (DPBF) assay and ESR were employed to qualitatively and quantitatively analyze the ^1^O_2_ generation by HMME@Lips, respectively. With the prolonging of US irradiation duration and the increase of HMME@Lips concentration, the characteristic absorbance intensity of DPBF decreases distinctly, suggesting that DPBF has been oxidized by the generated ^1^O_2_ (Figure 2d,e). The efficient ^1^O_2_ production was also quantitatively confirmed by ESR, in which characteristic 1:1:1 ^1^O_2_ signals can be detected, and the spin concentration of ^1^O_2_ radicals shows an HMME@Lips concentration‐dependent manner (Figure 2f). Based on the above results, it could be clearly observed that both Cu‐LDH and HMME alone can generate a large panel of ROS in vitro under certain conditions.

The production of ROS by Cu‐LDH/HMME@Lips at the cellular level was also systematically explored. Herein, 2′,7′‐dichlorofluorescein diacetate (DCFH‐DA), a fluorescent ROS indicator which can be oxidized by ROS and then emits green fluorescence, was employed to monitor the ROS level in cells after treated by different conditions. It can be seen in the flow cytometry analysis of ROS level in cells (Figure 2g) that 4T1 cancer cells treated with Cu‐LDH/HMME@Lips + US exhibit significantly stronger green DCFH signal than those treated with Cu‐LDH‐only or HMME‐only and other control cells. In addition, the consistent ROS‐associated fluorescence change was visualized under confocal laser scanning microscopy (CLSM, Figure S2, Supporting Information) after different treatments wherein 4T1 cells were treated with Cu‐LDH/HMME@Lips + US show the brightest green fluorescence. These data evidence that the designed ROS generator is highly efficient to generate ROS in tumor cells upon US irradiation.

**Figure 3:**
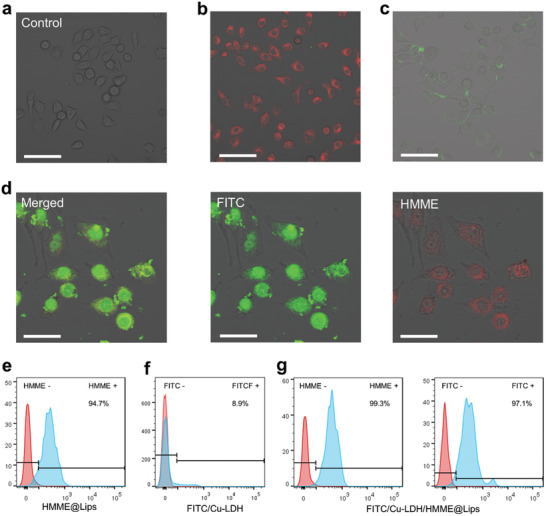
CLSM images of a) 4T1 cells without treatment (scale bar: 80 µm), b) after incubation with HMME@Lips for 20 min (scale bar: 80 µm), c) after incubation with FITC‐Cu‐LDH for 20 min (scale bar: 80 µm) and d) after incubation with FITC‐Cu‐LDH/HMME@Lips for 20 min (scale bar: 40 µm) showing images of the merged, FITC and HMME channels from the left to the right. e–g) Flow cytometry analyses of cellular uptakes of HMME in 4T1 cells after incubation with HMME@Lips for 20 min compared to that of e) control, f) FITC labeled Cu‐LDH in 4T1 cells after incubation with FITC labeled Cu‐LDH for 20 min, and HMME (left) and FITC‐labelled Cu‐LDH (right) g) in 4T1 cells after incubation with FITC‐Cu‐LDH/HMME@Lips for 20 min.

The effective cellular internalization of smart ROS generators, which is critical to trigger relevant therapeutic effects, was qualitatively and quantitatively validated by CLSM and flow cytometry, respectively. After co‐incubation with HMME@Lips for 20 min, intensive red fluorescence of HMME was observed in 4T1 cells compared to those in the control group (**Figure** **3**a,b). Correspondingly, the strong characteristic HHME signals were also detected by flow cytometry in 20 min incubation with HMME@Lips (Figure 3e). Those results indicate that liposoluble and negatively charged HMME@Lips could be internalized by cells rapidly, showing its great potential for mediating positively charged Cu‐LDH entry into cells. In contrast, both CLSM observation and flow cytometry detection indicate that cells fail to effectively uptake non‐capsulated positively‐charged FITC‐Cu‐LDH within such a short incubation period (Figure 3c,f). In fact, the easy aggregation of the non‐capsulated LDH nanosheets in physiological solution has stopped the rapid entry of positively charged Cu‐LDH into tumor cells, which is also a common obstacle encountered by other positively charged nanoparticles.^[^ ^7^, ^16^ ^]^ In sharp contrast, the strong HMME red and FITC green fluorescence emissions were observed in 4T1 cells after incubation with FITC‐Cu‐LDH/HMME@Lips for 20 min, demonstrating the efficient internalization of ROS generators (Figure 3d). Additionally, this result is further evidenced by the flow cytometry analysis wherein the strong intracellular characteristic HMME and FITC signals were detected in 20 min co‐incubation (Figure 3g). Collectively, the designed ROS generators can be rapidly internalized by tumor cells, owing to the negatively charged liposome covering the initially positively charged Cu‐LDH, thereby enabling the efficient cellular internalization of the catalyst Cu‐LDH for the following solid tumor therapy in vivo.

**Figure 4:**
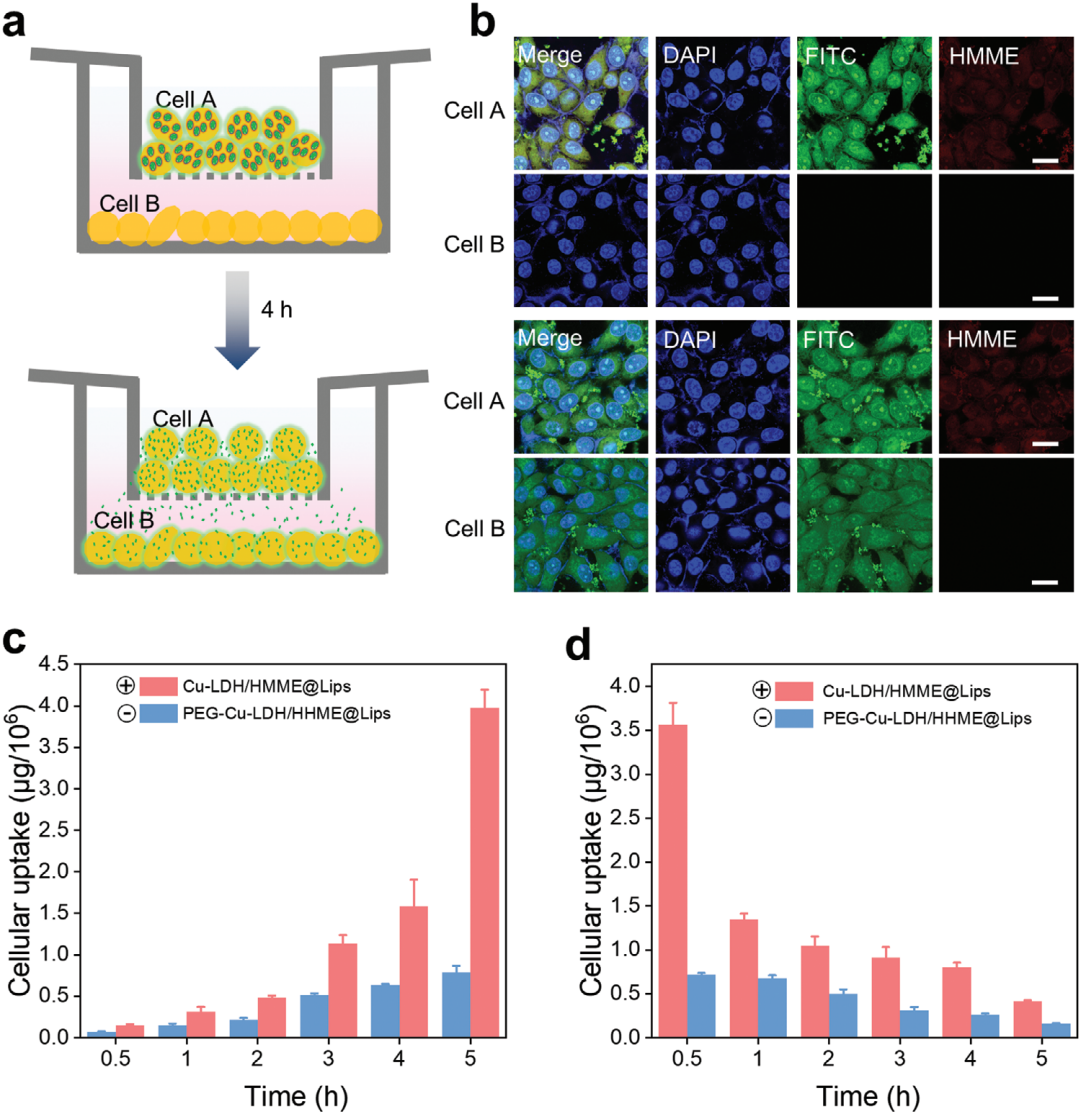
a) Suspension of Cu‐LDH/HMME@Lips‐encapsulated cells (cell A) was added into the upper compartment (0 h) and then incubated for 4 h under US irradiation leading to the disruption of the initial nanoplatforms (larger green circles) and the release of Cu‐LDH (smaller green points). b) CLSM images of cell A and cell B in the left migration model in 0 h (upper two) and 4 h (bottom two)wherein the small green dots present the inevitably aggregated FITC‐labeled Cu‐LDH in/at the cells. Scale bar: 50 µm. c) The internalized doses of in 4T1 cells after incubations respectively with Cu‐LDH/HMME@lips and PEG‐Cu‐LDH/HMME@lips for varied periods. d) The remaining doses of 4 h pre‐endocytosed Cu‐LDH and PEG‐Cu‐LDH in 4T1 cells after the following cultivation in fresh DMEM for different periods.

Transcytosis has been recently reported to be favorable for the deep intratumoral penetration of nanomedicines, especially for positively charged ones. Transcytosis is a special type of endocytosis of cells, during which the nanoparticles can be completely uptaken into the cell but then released from the cells via exocytosis.^16^ To evaluate the transfer capability of the designed ROS generator in between cells, we established a nanomedicine migration model (**Figure** **4**a) by seeding 4T1 cells in both upper and bottom compartments of a nested Transwell culture system, in which the microporous (1 µm in diameter) polyester membrane can block cells but not nanoparticles. In this scenario, 4T1 cells pre‐incubated with FITC‐Cu‐LDH/HMME@Lips (cells A) were added to the upper compartment, while blank cells (cells B) were pre‐adhered to the bottom compartment. As displayed in Figure 4b, the intensive green FITC‐Cu‐LDH and red HMME fluorescence signals can only be observed in cells A but not in cells B before co‐incubation. After US irradiation for 5 min allowing the FITC‐Cu‐LDH/HMME@Lips to rupture and followed incubation for 4 h, green FITC‐Cu‐LDH fluorescence could also be detected in cells B, demonstrating that the endocytosed positively charged FITC‐Cu‐LDH mediated by liposomes has been partially released from cells A into the upper medium, crossed over the membrane, and then been uptaken by cells B in the bottom compartment. Comparatively, the negatively charged HMME failed to transfer between cells in such a short period, as confirmed by the negligible red HMME fluorescence in cells B. Furthermore, the effective internalization and excretion dynamic processes of Cu‐LDH into/from the cells were also in‐situ observed by Bio‐TEM after the cells were co‐incubated with Cu‐LDH@Lips (Figure S3, Supporting Information).

The cellular internalization and excretion of Cu‐LDH/HMME@Lips were further quantified to offer strong evidence that the positively charged Cu‐LDH is more favorable for the deep intratumoral penetration via transcytosis than the negative one. In this assay, Cu‐LDH modified with polyethylene glycol (PEG‐Cu‐LDH/HMME@Lips), which was finally negatively charged (Table S2), was applied as a control. The intracellular accumulation amount of Cu‐LDH is approximately twice that of PEG‐Cu‐LDH at the first five incubation time points, and even reached 5 times in 5 h incubation wherein the final uptake dose is as high as ≈3.97 µg 10^−6^ cells for Cu‐LDH in comparison to ≈0.79 µg 10^−6^ cells for PEG‐Cu‐LDH (Figure 4c). Next, we incubated those nanoparticle‐internalized 4T1 cells in a fresh culture medium for different time durations and measured the remaining intracellular doses in different time intervals to evaluate the exocytosis of Cu‐LDH. It can be found that the residual intracellular amounts of positive Cu‐LDH are significantly higher than those of negative PEG‐Cu‐LDH. Noticeably, after cultivation for 5 h, the exocytosis dose of Cu‐LDH was approximately 0.32 µg 10^−6^ cells, which is ∼4.6‐fold higher than that of PEG‐Cu‐LDH (≈0.056 µg 10^−6^ cells) (Figure 4d). These results indicate that with the assistance of liposomes, the released positively charged Cu‐LDH could migrate more efficiently from one tumor cell to the other in a short time interval than the negatively charged counterpart, which enables its further deep penetration inside the tumor.

Subsequently, the in vivo biological behavior and tumor permeability of the nanocatalyst was further investigated. The blood‐circulation half‐life of Cu‐LDH@Lips was calculated to be 3.42 h following a two‐compartment model, while the that of free Cu‐LDH was much shorter at 1.08 h (Figure S4, Supporting Information). This distinct difference in blood circulation half‐lives between Cu‐LDH@Lips and Cu‐LDH manifests that with the protection of liposomes, the positively charged catalyst can be effectively prevented from rapid clearance in vivo. The prolonged blood‐circulation provide sufficient time durations for catalyst Cu‐LDH to effectively accumulate inside tumor via EPR effect, subsequently achieving deep intratumoral penetration via transcytosis. Furthermore, the quantitative measurement of the biodistribution also demonstrates that the tumor passive‐targeting efficiency of Cu‐LDH@Lips in 4 h post‐injection is much higher than that of free Cu‐LDH (9.15% v. s. 2.32%, Figure S5, Supporting Information).

**Figure 5:**
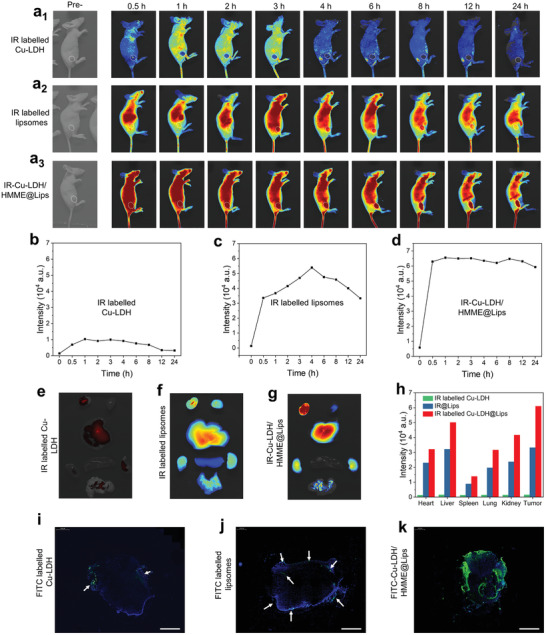
Real‐time fluorescence images of solid 4T1 tumor‐bearing mice before and after the intravenous injections of a_1_) free IR labeled Cu‐LDH, a_2_) IR labeled liposomes, and a_3_) IR labeled Cu‐LDH@Lips. Real‐time fluorescence intensities at the tumor sites of mice after the intravenous injections of b) IR labeled Cu‐LDH, c) IR labeled liposomes, and d) IR‐Cu‐LDH/HMME@Lips. Ex vivo fluorescence images of main organs (liver, spleen, lung, heart, kidney) and tumors, which were obtained 24 h post‐injections of e) IR‐Cu‐LDH/HMME@Lips, f) IR‐labelled liposomes, and g) fresh IR‐labelled Cu‐LDH. h) Fluorescence intensities of liver, spleen, lung, heart, kidney, and tumor after treated with IR‐Cu‐LDH/HMME@Lips, IR labeled Cu‐LDH and IRlabelled liposomes for 24 h. i–k) Penetration overviews of FITC‐labelled Cu‐LDH, FITC‐labelled liposomes, and IR‐Cu‐LDH/HMME@Lips in the whole tumor, scale bar 2 mm.

Moreover, we carried out the fluorescence imaging assay by respectively injecting IR‐Cu‐LDH/HMME@Lips and IR783‐labelled Cu‐LDH nanoparticles into mice model (20 mg kg^−1^, 100 µL) to track theirs in vivo behaviors. Meanwhile, the mice injected with pure IR783‐labelled liposomes were employed as control, wherein IR783 acted as a fluorescent molecule emitting 810 nm red light under the excitation at 780 nm. Only a rather weak fluorescence signal can be observed in nude mice treated with IR783‐labelled Cu‐LDH (**Figure** **5**a1), corresponding to the negligible fluorescent intensity at the tumor site (Figure 5b). This result suggests that the small positively charged and easily agglomerated Cu‐LDH is difficult to circulate in the body, not to mention to accumulate inside tumor effectively. Much stronger fluorescence in vivo and a transient plateau of fluorescence intensity at the tumor site of mice in 4 h can be seen in the group injected with IR783 labeled liposomes (Figure 5a2,c), elucidating that the negatively charged and rather long‐circulatory liposomes of ≈200 nm in size could achieve more effective EPR‐derived tumor accumulation than Cu‐LDH. By comparison, the strongest fluorescence signal and long‐lasting fluorescence intensity plateau at tumor site could be obtained in the IR‐Cu‐LDH/HMME@Lips group, owing to the size and charge switching of Cu‐LDH/HMME@Lips upon US irradiation after accumulation in tumor tissue (Figure 5a3,d). The *ex vivo* imaging of main organs (liver, spleen, lung, heart, and kidney) and tumors of mice in 24 h of intravenous injection was also performed. Among the experimental groups tested, the tumor tissue in IR‐Cu‐LDH/HMME@Lips group shows the strongest fluorescence signal and intensity (Figure 5e–h). In addition, to more intuitively demonstrate the intratumoral permeability of different nanoparticles, the whole tumors were harvested after the injections of FITC labeled Cu‐LDH, FITC labeled liposomes and FITC labeled Cu‐LDH/HMME@Lips for 4 h. Then, these tumors were sliced to visualize the distribution of FITC green fluorescence in the tumor under CLSM. The result shows that rather weak green fluorescences of FITC labeled Cu‐LDH and FITC labeled liposomes can be observed in the tumor with slightly stronger fluorescence being detected at the tumor edges (depth of 0.11 and 0.21 mm, respectively) due to either the insignificant accumulation at the tumor site or poor intratumoral infiltration. However, far brighter fluorescence of FITC labeled Cu‐LDH/HMME@Lips can be found in a quite large area of the tumor (depth of 2.13 mm), which proves that Cu‐LDH/HMME@Lips possesses the strongest tumor permeability in addition to the effective accumulation at the tumor site (Figure 5i–k, Figure S6, Supporting Information). Overall, it has been evidenced that the designed Cu‐LDH/HHME@Lips presents a relatively long circulatory half‐life to achieve effective tumor accumulation, and the positively charged small Cu‐LDH nanosheets can be then released from the liposome micelles upon US irradiation for deep intratumoral penetration following the accumulation in tumor tissue.

Based on the effective deep tumor penetration performance and excellent ROS generation ability of Cu‐LDH/HMME@Lips, we expect that the nanoplatform could trigger powerful anti‐cancer effects both in vitro and in vivo. Thus, the cell counting kit‐8 (CCK‐8) assay was initially performed to evaluate the anticancer efficiency of Cu‐LDH/HMME@Lips in vitro. The in vitro anticancer efficacy becomes more significant at the increased Cu‐LDH concentrations, but the cytotoxicity of Cu‐LDH is negligible when intracellular H_2_O_2_ has been pre‐consumed by catalase (CAT), suggesting that intracellular H_2_O_2_ is a prerequisite for its nanocatalytic anti‐cancer effect (Figure S7a, Supporting Information). In addition, the sonosensitizer HMME could effectively generate ROS to kill 4T1 cells upon US irradiation compared to the HMME‐only or US‐only treatments (Figure S7b, Supporting Information). Moreover, since the external energy input, which can be regarded as an external catalyst in general, can catalyze the reaction of Cu‐LDH and H_2_O_2_, the cytotoxicity responses of Cu‐LDH is substantially elevated upon external US irradiation (Figure S7c, Supporting Information). In all, the constructed ROS generator Cu‐LDH/HMME@Lips exhibits the most marked anti‐cancer effect in vitro after US irradiation. Then, the living and dead cells after different treatments were visualized by a calcein acetoxymethyl ester (Calcein‐AM)/propidium iodide (PI) double staining assay. Only bright green calcein‐AM fluorescence signals can be observed in the control, HMME, Cu‐LDH@Lips + CAT, and US groups, which manifests that those treatments cannot effectively kill 4T1 cells. Strong red PI fluorescence can be seen in the Cu‐LDH/HMME@Lips + US group, while both green and red fluorescence can be found in Cu‐LDH@Lips, Cu‐LDH@Lips +US, and HMME@Lips + US groups, confirming that engineered ROS generator demonstrates the remarkable tumor cell killing effect in vitro (Figure S7d, Supporting Information). Similar results were likewise obtained by the flow cytometry analysis via fluorescein isothiocyanate (FITC)‐labeled annexin V and PI staining. Obviously, the most significant cancer cell apoptosis has been achieved in the Cu‐LDH/HMME@Lips + US treatment group among all groups tested (Figure S7e, Supporting Information).

The human umbilical vein endothelial cells (HUVECs) was also used in this experiment for comparison. Single Cu‐LDH/HMME@Lips treatment has a negligible effect on the survival of HUVECs, due to the lack of sufficient peroxidase substrate H_2_O_2_ in HUVECs (Figure S8, Supporting Information). The ultrasound‐enhanced nanocatalytic therapy shows high therapeutic specificity in response to the external stimuli, which accelerates the chemical reactions (such as Fenton‐like reactions) only at the tumor target but normal cells/tissues. This specific anticancer behavior of LDH/HMME@Lips ensures tumor‐specific therapy and bio‐safety in further application in vivo.

Subsequently, we systematically investigated the biocompatibility of Cu‐LDH/HMME@Lips. Healthy BALB/c mice were randomly divided into four groups (*n* = 5) and injected intravenously with PBS, HMME@Lips, Cu‐LDH@Lips (20 mg kg^−1^), and Cu‐LDH/HMME@Lips (20 mg kg^−1^), respectively. During the one month observation period, the body weight of mice in four groups did not show any apparent decrease (Figure S9a, Supporting Information). Moreover, the blood and major organs (heart, liver, spleen, lung, kidney) were collected from the treated mice for further evaluation at the end of observations. The hepatic function indexes (ALT, AST, ALP) of mice in different groups were measured to be normal after being treated with different materials, indicating the negligible liver dysfunction by the designed Cu‐LDH/HMME@Lips (Figure S9b, Supporting Information). Also, other blood indexes of mice in HMME@Lips, Cu‐LDH@Lips, and Cu‐LDH/HMME@Lips groups present no significant abnormal compared with the control group (Figure S9c, Supporting Information). Hematoxylin and eosin (H&E) staining of main organs of mice in each group shows negligible tissue abnormalities or damages, further confirming the excellent biocompatibility and high biosafety of the designed Cu‐LDH/HMME@Lips in vivo (Figure S9d, Supporting Information).

**Figure 6:**
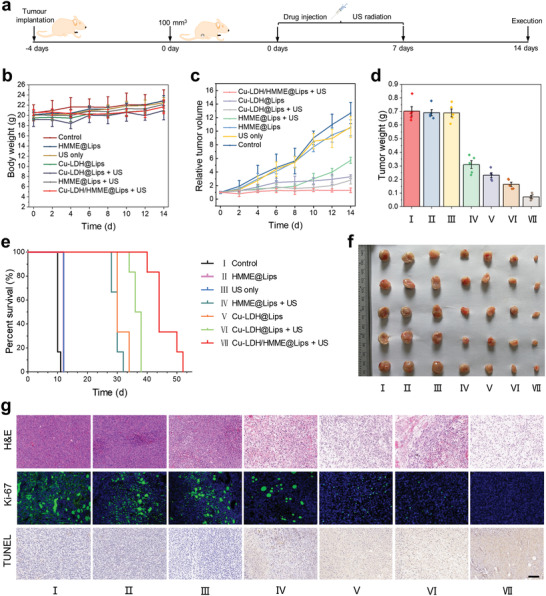
In vivo tumor therapeutic effect of Cu‐LDH/HMME@Lips on 4T1 tumor‐bearing mice. a) Schematics of the establishment of 4T1 tumor‐bearing mouse model and in vivo treatment process. b) Body weights of mice after various treatments. c) Time‐dependent relative tumor volumes change after various treatments and f) the final tumor weight of mice in different groups. e) Kaplan–Meier survival curves of 4T1 tumor‐bearing mice in the different groups. f) Digital images of tumors acquired from the mice in different groups at the end of treatments. g) H&E staining, Ki‐67 immunofluorescence labeling, and TUNEL staining images of 4T1 xenograft tumor sections in 12 h after different treatments (scale bar: 200 µm).

The significant in vitro therapeutic outcomes and desirable biocompatibility of Cu‐LDH/HMME@Lips encouraged us to extensively explore its antitumor effect in vivo. Initially, 4T1 breast cancer cells were injected subcutaneously into the right legs of female Balb/c nude mice to establish a subcutaneous tumor model for evaluation. When the tumor volume reached approximately 100 mm^3^, 4T1 tumor‐bearing mice were randomly divided into seven groups and treated with: I) control; II) HMME@Lips; III) US only; IV) HMME@Lips + US; V) Cu‐LDH@Lips; VI) Cu‐LDH@Lips + US; VII) Cu‐LDH/HMME@Lips + US, respectively (**Figure** **6**a). During a treatment period of 14 days, the body weights and tumor volumes of the mice were recorded every two days. Moreover, the main organs (heart, liver, spleen, lung, kidney) and tumor tissues of mice were harvested and stained with H&E for pathological analysis. No significant body weight fluctuations and pathological changes of mice have been observed in all groups, indicating the negligible harmful effect on the mice's health of these treatments (Figure 6b, Figure S10, Supporting Information). It is worth noting that the tumor in mice kept rapid growth in the control, US‐only, and HMME@Lips groups. Comparatively, the tumor growth could be partially suppressed after the mice were treated with Cu‐LDH@Lips, HMME@Lips + US, or Cu‐LDH@Lips + US. Notably and importantly, Cu‐LDH/HMME@Lips + US treatment exhibits the most pronounced antitumor effect, where the tumor suppression rate has been calculated to be 89.7%, confirming that the designed deep therapy strategy could realize superadditive therapeutic efficacy (Figure 6c,d,f). Furthermore, mice in the Cu‐LDH/HMME@Lips + US group completely kept alive in 40 days after therapy, while all mice in the other six groups gradually died owing to the high invasiveness of the tumors (Figure 6e). H&E and terminal deoxynucleotidyl transferase uridine triphosphate nick end labeling (TUNEL) staining results reveal that much more significant tumor‐cell apoptosis or necrosis in the Cu‐LDH/HMME@Lips + US group compared with other treatment groups (Figure 6g). Meanwhile, Ki‐67 antibody staining was performed to assess cell proliferation, which exhibits excellent suppression effect on tumor cell proliferative activity in Cu‐LDH/HMME@Lips + US group (Figure 6g).

To uncover the antitumor mechanism of Cu‐LDH/HMME@Lips + US, the intratumoral ROS level after various treatments were explored by an in vivo ROS staining assay in which DCFH‐DA was used as a fluorescent probe (Figure S11, Supporting Information). Weak green DCF fluorescence signals were observed in the control, US, and HMME@Lips treatment groups due to ROS overexpressing in the tumor tissue. And the fluorescence signal becomes slightly brighter after treated by Cu‐LDH@Lips, Cu‐LDH@Lips + US, and HMME@Lips + US, indicating that the intratumoral ROS levels have been elevated by the nanocatalytic therapy. Notably, as‐designed ROS generators capable of deep tumor‐infiltrating has triggered a great amount of ROS generation upon US irradiation, as indicated by the strong green DCF fluorescence signal. Therefore, it is the increased intratumoral ROS level by the designed ROS generator that should be responsible for the valid anti‐cancer effect.

## 3 Conclusion

In this work, efforts have been dedicated to constructing a sequentially responsive size/charge‐switchable ROS generator, designated as Cu‐LDH/HMME@Lips, wherein liposomes act as a carrier to co‐encapsulate catalytic medicine Cu‐LDH and sonosensitizer HMME. The relatively large and negatively charged carrier endows the generator with a long blood circulation duration for tumoral accumulation. After the accumulation, Cu‐LDH/HMME@Lips can rapidly disassemble themselves to liberate the encapsulated, size‐reduced, and positively charged Cu‐LDH nanosheets upon US irradiation, concurrently achieving size and charge switching, and meanwhile the generation of ^1^O_2_ species from the US‐stimulated HMME. Resultantly, the released positively charged Cu‐LDH catalyst can infiltrate deeply into the tumor tissue through transcytosis, subsequently generate highly toxic •OH by catalyzing the decomposition of the over‐expressed H_2_O_2_ under the specific mildly acidic TME. Consequently, the cellular redox balance is disrupted by the significantly increased ROS (^−^O_2_, •OH), leading to the apoptosis of cancer cells and the ultimate tumor growth suppression in vivo. This investigation offers a promising perspective on the development of nanocatalytic medicines to improve chemo‐dynamic therapy efficacy against solid tumors.

## Conflict of Interest

The authors declare no conflict of interest.

## Supporting information

Supporting Information
