# Supplementary material for: Dual Size/Charge‐Switchable Nanocatalytic Medicine for Deep Tumor Therapy
Source: Adv Sci (Weinh). 2021 Mar 1;8(9):2002816. doi: 10.1002/advs.202002816 (PMC8097343; doi:10.1002/advs.202002816)
Supplement: Supplementary file 1 — Supporting Information [file ADVS-8-2002816-s001.pdf]

© 2020 Wiley-VCH GmbH

Supporting Information

for *Adv. Sci.*, DOI: 10.1002/advs.202002816

Dual Size/Charge-Switchable Nanocatalytic

Medicine for Deep Tumor Therapy

*Wencheng Wu, Yinying Pu, and Jianlin Shi**

Supporting Information

Dual Size/Charge-Switchable Nanocatalytic Medicine for Deep Tumor Therapy

*Wencheng Wu, Yinying Pu, and Jianlin Shi**

A: Experimental Section

1. Materials

$MgCl_2·6H_2O$, $AlCl_3·6H_2O$, NaOH and $CuCl_2·2H_2O$, were acquired from Adamas-Beta Co. (Shanghai, China). Dipalmitoyl phosphatidylcholine (DPPC), 1,2-distearoyl-sn-glycero-3-phosphoethanolamine-N-[amino(polyethylene glycol) 2000] (DSPE-PEG-2000), cholesterol were obtained from Xi'an Ruixi Biological Technology Co. (Xian, China). Phosphate buffer solution (PBS) were purchased from Sinopharm Chemical Reagent Co. (Shanghai, China). Hematoporphyrin monomethyl ether (HMME), 1,3-diphenylisobenzofuran 1,3 (DPBF), Dimethyl sulfoxide (DMSO), and 3,3',5,5'-Tetramethylbenzidine (TMB) were purchased from Sigma-Aldrich Co. (Shanghai, China). Dulbecco's modified eagle medium (DMEM high glucose, United Bioresearch, Inc). Calcein, 4, 6-diamidino-2-phenylindole (DAPI), propidium iodide (PI) and were ordered from JenKem Technology Co. (Beijing, China). Fluorescein isothiocyanate (FITC) and cell counting Kit-8 (CCK-8) were purchased from Beyotime Biotechnology Co. (Haimen, China).

2. Fabrication of fresh copper doped magnesium aluminum layered double hydroxide (Cu-LDH)

Cu-LDH was obtained by Cu-substitution on fresh precursor Mg_3Al-LDH. First, original Mg_3Al-LDH nanosheets (LDHs) synthesized using a solvent-free bottom-up method. Typically, $MgCl_2$ (0.6 M) and $AlCl_3$ (0.2 M) were premixed with deionized water (5 mL) and then rapidly were added into NaOH solution (20 mL, 0.4 M). After stirring vigorously for 15 min, the fresh Mg_3Al-LDH products were collected by centrifugation (19000 rpm, 20 min), purified by deionized water three times, and re-suspended in deionized water (20 mL) for further use. In the second step, the prepared fresh Mg_3Al-LDH suspension (2 mL) was blended with aqueous $CuCl_2·2H_2O$ solution (20 mL, 0.1 M) with Ar bubbling under stirring at 25 ℃ for 6 h. The resultant product was separated by centrifugation (19000 rpm, 20 min) and rinsed using deionized water three times to remove dissociative Cu^{2+}.

3. Preparation of Cu-LDH/HMME@Lips.

Cu-LDH/HMME@Lips were synthesized via a thin-film hydration method. Briefly, HMME and Cu-LDH were firstly dissolved in methanol (6 mg/ml) and PBS (4 mg mL^{-1}) for further use, respectively. Afterward, HMME (0.1 ml) solution was added into the trichloromethane organic solvent, which containing DPPC, DSPE-PEG-2000, and cholesterol at a fixed weight ratio of 3:1:1. The trichloromethane was removed by the following evaporation on a rotary evaporator at 60 °C for 1 h, and the resultant thin films were dried overnight under vacuum. Then, 5 ml PBS containing Cu-LDH (4 mg mL^{-1}, pH = 7.4) was added into the flask, and the mixture was rotated for another 1 h to fully emulsify the thin film. Finally, the vesicles were obtained by a repeated extrusion process on a high-pressure extruder (ATS Engineering Inc, Jiangsu, China) using 1000- and 400- nm membranes, respectively, and then further purified by dialysis.

4. Characterization

The hydrodynamic size distribution and zeta potential were determined by a Malvern Zetasizer Nano series (Malvern Panalytical, Malvern, UK). X-ray diffraction measurements (XRD Bruker D8 Focus, Bruker, Billerica, MA, USA; 2θ ranging from 10° to 90° Cu $K_{\alpha1}$) were performed on as-synthesized fresh LDH and Cu-LDH powder. At an accelerating voltage of 200 kV, transmission electron microscopy (JEM-2100F, Tokyo, Japan) and scanning electron microscopy (JEOL Ltd., Tokyo, Japan) imaging coupled and energy-dispersive X-ray spectroscopy (EDS) elemental analysis were applied to capture their morphology and chemical components, respectively. The valence of copper doped in LDH was analyzed from the X-ray photoelectron spectroscopy (XPS) spectrum which gets by a Thermo ESCALAB250i spectrometer (Thermo Fisher Scientific, Waltham, MA, USA). Electron spin resonance (ESR) measurements were performed on a jeol-fa200 spectrometer at room temperature with the following settings: microwave frequency = 9.425 GHz, microwave power = 0.998 mW, modulation frequency = 100.00 kHz, and modulation amplitude = 2.00 G. DMPO and TEMP were used as the spin trap of ·OH and $^1O_2$, respectively. All UV−Vis absorption spectra were obtained from a UV-3600 Shimadzu spectrometer. And all the quantitative element analysis was determined by inductively coupled plasma optical emission spectrometer (ICP-OES, Agilent

Technologies, US). The encapsulation efficiency of HMME in the Lips was measured by UV-vis spectra technology. The amount of Cu-LDH encapsulated in the Lips was determined by ICP-OES.

5. Detection of ·OH and $^1O_2$ *in vitro*

TMB assay was performed to monitor the chromogenic reaction of the Cu-LDH/H_2O_2 system. In PBS (800 μL) with different pH values (7.4, 5.0), Cu-LDH suspensions (0, 4, 8, 16, 32 μg mL^{-1}) were mixed with TMB and H_2O_2 at the final concentrations of 800 μM and 20 mM, respectively. Conversely, in PBS (800 μL) with different pH values (7.4, 5.0), H_2O_2 suspensions (0, 2, 5, 10, 20 mM) were mixed with TMB and Cu-LDH at the final concentrations of 800 μM and 32 μg mL^{-1}, respectively. The absorbance (λ = 650 nm) of the mixture was measured by a UV-Vis spectrometer. And as control groups, LDHs/H_2O_2 system was also detected. For detection of the generation of $^1O_2$, HMME@Lips was suspended in PBS (pH = 7.4, 200 μg mL^{-1}), followed by adding DPBF (40 μL, 8mM). Then, the absorbance intensity of the mixture was recorded using a fluorescence spectrometer after upon exposure to US irradiation (1.0 MHz, 1.5 W/cm2, 50% duty cycle) every 1 min in dark. Complementarily, the absorbance intensity of mixture with different concentrations of HMME@Lips (0, 25, 50, 100, 200 μg mL^{-1}) also was detected after irradiating by US for 5 min.

As for the quantitative analysis of ·OH, four groups were setted as followed: H_2O_2 (100 μM) only, Cu-LDH@Lips (pH = 7.4) + H_2O_2, Cu-LDH@Lips (pH = 5.0) + H_2O_2, and Cu-LDH@Lips (pH = 5.0) + H_2O_2 + US, all group shared the equivalent concentration of Cu-LDH of 32 μg mL^{-1} (200 μL). Immediately after the addition of DMPO (5 μL), the ·OH generation was detected by an ESR spectrometer. In addition, the quantitative generation of $^1O_2$ is based on the varied concentration of Cu-LDH/HMME@Lips (at the equivalent HMME concentrations of 0, 50, 100, and 200 μg mL^{-1}) were also measured by an ESR spectrometer.

6. Intracellular ROS detection

4T1 cells were treated with HMME@Lips (200 μg mL^{-1}), US, HMME@Lips (200 μg mL^{-1}) + US, Cu-LDH@Lips (Cu^{2+}: 20 μg mL^{-1}), Cu-LDH@Lips, Cu-LDH@Lips + CAT, Cu-LDH@Lips + US, and Cu-LDH/HMME@Lips + US. After incubation for 8 h, the cells were

washed with PBS three times and stained by 2, 7-dichlorodihydrofluorescein diacetate (DCFH-DA) which is a ROS fluorescent probe. Finally, the intracellular ROS signal was observed by CLSM and detected by flow cytometry.

7. *In vitro* cellular-uptake

4T1 cells were cultivated in confocal dishes (2×10^5 cells per well) for 12 h. Then, the cells were incubated with free FITC labeled Cu-LDH, HMME@Lips, FITC @Lips, and FITC-Cu-LDH/HMME@Lips for 20 min, respectively. After washing with PBS three times to move the residual materials, the cellular uptake was monitored by CLSM and quantitatively detected by flow cytometry.

8. Cellular internalization and excretion of Cu-LDH

4T1 cells were seeded into a 12-well plate overnight to allow cells to adhere. Then, the cells (n = 3) were incubated with Cu-LDH@Lips and PEG-Cu-LDH@Lips for different times (0, 0.5, 1, 2, 3, 4, and 5 h), respectively. After washed with PBS, the cells were then digested, resuspended, and counted. The metal contents (Cu, Mg, and Al) in the cell suspensions were determined by ICP-OES. The intracellular contents of Cu-LDH were calculated accordingly.

In the excretion process, 4T1 cells (n = 3) that had been incubated with Cu-LDH@Lips (200 μg mL^{-1}) and PEG-Cu-LDH@Lips for 5 h were recultivated in fresh DMEM for varied times (0, 0.5, 1, 2, 3, 4, and 5 h). At each time point, cells were harvested using trypsin after washed with PBS 3 times, resuspended, and counted. The remaining contents of Cu-LDH in cells were determined by ICP-OES. The internalization and excretion of Cu-LDH nanosheets in cells were further monitored by bio-TEM directly. Typically, the ultrathin sections of 4T1 tumor cells were made after coincubated with Cu-LDH/HMME@Lips for varied times (2, 4, and 8 h) and *in-situ* observed by bio-TEM directly.

9. Migration of Cu-LDH/HMME@Lips in a transwell system

To observe the migration of Cu-LDH with positive zeta potential and HMME with negative zeta potential between 4T1 cells, a migration model in a transwell system (Corning, USA) was established. A 3 μm-diameter microporous polyester membrane was used to block the migration of cells. In this case, blank 4T1 cells were seeded onto the basolateral compartment of the

transwell system and cultured for overnight to allow cell adhesion. Additional blank cells were firstly incubated with FITC labeled Cu-LDH/HMME@Lip for 4 h. After washing with PBS 3 times, cells were digested and added back to the apical compartment and US irradiation for 5 min and followed by incubation for another 4 h. Finally, DAPI was used to stain the cell nucleus and was observed by multiphoton CLSM.

10. *In vitro* antitumor activity

For *in vitro* antitumor activity evaluation, 4T1 cells were plated in 96-well plates (1×10^5 cells per well) and cultured for 24 h. In the blank HMME@Lips group, the cells were treated with HMME@Lips (0, 12.5, 25, 50, 100, and 200 μg mL^{-1}) in 100 μL of complete DMEM. To prove the indispensability of H_2O_2 in the action of Cu-LDH@Lips, 4T1 cells were incubated with Cu-LDH@Lips (Cu^{2+}: 0, 1.25, 2.5, 5, 10, and 20 μg mL^{-1}) in 100.0 μL of complete DMEM or pre-treated with catalase (CAT) before incubation with Cu-LDH@Lips. The sonotoxicity against cancer cells was also further evaluated. 4T1 cells were initially incubated with Cu-LDH@Lips, Cu-LDH/HMME@Lips, or HMME@Lips for 12 h. Subsequently, they were exposed to US irradiation (1.0 MHz, 1.5 W cm^{-2}, 50% duty cycle, 1 min) and then incubated for another 12 h. After incubation, all of the relative cell viability was measured using the standard CCK-8 assay based on the absorbance at the wavelength of 450 nm.

For observations by CLSM, 4T1 cells (1×10^5 cells) were dispersed on a CLSM-exclusive culture disk (φ = 15 mm, Corning Inc., NY, USA), and incubated for 12 h to facilitate adherence of cells. Then, 4T1 cells were treated as followed group described for 24 h: HMME@Lips (200 μg mL^{-1}), US, HMME@Lips (200 μg mL^{-1}) + US, Cu-LDH@Lips (Cu^{2+}: 20 μg mL^{-1}), Cu-LDH@Lips, Cu-LDH@Lips + CAT, Cu-LDH@Lips + US, and Cu-LDH/HMME@Lips + US. After that, the cells were stained by Calcein-AM/PI, followed by observation using CLSM. For flow cytometry analysis, 4T1 cells were dispersed in six-well microplates (1×10^5 cells/plate) and treated as described above. Prior to analysis, cells were harvested using trypsin and re-suspended in Annexin-binding buffer (200 μL) and washed twice with PBS. Then, FITC (5 μL) and PI (5 μL) were added to the buffer staining solution and incubated for 0.5 h in the dark. Stained cells were final analyzed using a BD LSRFortessa flow cytometer (Becton, Dickinson and Company, USA).

11. Animal research

Female Kunming mice (8 weeks) and BALB/c nude mice (6 weeks) were purchased from Shanghai SLAC Laboratory Animal Company. All experimental protocols were approved by the Laboratory Animal Center of Shanghai Tenth Peoples' Hospital and complied with the policies of the National Ministry of Health.

12. *In vivo* blood circulation of Cu-LDH@Lips and free Cu-LDH

Cu-LDH@Lips and free Cu-LDH in saline solution (100 μL, 4 mg mL^{-1}) were intravenously injected into mice (n = 3), and equal blood (20 μL, n = 3) was sampled at given time points (2, 5, 10, 20, and 30 min, 1, 2, 4, 8, 12, and 24 h). Then, the Mg contents in blood samples after treating by chloroazotic acid were analyzed by ICP-OES.

13. *In vivo* biodistribution

When tumor volumes of nude mice (n = 3) reached 200 mm^3, saline solution containing Cu-LDH@Lips (100 μL, 20 mg kg^{-1}) and saline solution containing PEG-Cu-LDH@Lips (100 μL, 20 mg kg^{-1}) was administered intravenously, respectively. The main organs (heart, liver, lung, spleen, kidney) and tumors of mice were harvested at 4, 12, and 24 h. Mg contents in organs and tumors were measured by ICP-OES after treated with chloroazotic acid.

14. *In vivo* toxicity study

The Kunming mice (~20 g, n = 5) were intravenously injected with pure saline (100 μL), saline containing HMME@Lips, saline containing Cu-LDH@Lips (20 mg kg^{-1}), and Cu-LDH/HMME@Lips (20 mg kg^{-1}) at the first and 15th day. The body weight of mice was measured every second day. At 30 days, blood of mice sampled by eyeball extirpating was used for hematological and biomedical indexes analysis. After mice were sacrificed, their major organs (heart, liver, spleen, lung, and kidney) were harvested and then fixed in a 10% formalin solution and embedded in paraffin for histopathology analysis using a typical hematoxylin and eosin (H&E) staining assay.

15. *In vivo* imaging and biodistribution analysis

When the volumes of the 4T1 tumors reached about 200 mm^3, the BABL/C mice were randomly divided into three groups and injected intravenously with free IR783 labeled Cu-LDH,

IR783 labeled liposomes, and IR783 labeled Cu-LDH@Lips (100 μL, 20 mg kg^{-1}) *via* the tail, respectively. Then, US irradiations in the above groups were performed in 4 h post-injection. The fluorescence signals of IR783 were detected by an *ex*/*in vivo* imaging system (VISQUE Invivo Smart-LF, Korea). The mice were sacrificed in 24 h after injection. The major organs (heart, liver, spleen, lung, kidneys) and tumors were collected for semiquantitative biodistribution analysis and imaging using the *ex*/*in vivo* imaging system.

16. *In vivo* anti-cancer effect evaluation

BALB/c nude mice were used to establish xenografted tumors models. In this experiment, tumors were planted by subcutaneously injecting 4T1 cells (1×10^7 cells suspended in PBS) into the mouse rear leg. Once tumor volumes reached 100 mm^3, mice were divided into 7 groups randomly (n = 7) including: (1) pure saline, (2) pure saline + US, (3) HMME@Lips, (4) HMME@Lips + US, (5) Cu-LDH@Lips, (6) Cu-LDH@Lips + US, (7) Cu-LDH/HMME@Lips + US. They were *i.v.* injected into animals at the same doses of HMME (14 mg kg^{-1}) on 0 and 7th day. US irradiations in the above groups were performed in 6 and 12 h post-injection. Their body weight and tumor volumes were monitored every other day after administration. At the end of the treatment, mice were sacrificed and their tumors were excised, weighed, and photographed. The pathological tissue sections of tumors were collected in 24 h post-treatment for H&E TUNEL and Ki-67 staining assay.

17. *In vivo* ROS evaluation

4T1 tumor-bearing mice were treated by pure saline, pure saline + US, HMME@Lips, HMME@Lips + US, Cu-LDH@Lips, Cu-LDH@Lips + US, and Cu-LDH/HMME@Lips + US, respectively. These mice were killed after injection for 12 h, and tumor tissues were immediately collected, and sectioned and DCFH-DA stained for CLSM observation.

18. Statistical analysis

Data were analyzed by OriginLab statistical software. Quantitative data are expressed as mean ± s.d. The sample size n for each group is 5. Statistical comparisons were conducted by using Student's two-sided t-test as $*P < 0.05$ (significant), $**P < 0.01$ (moderately significant) and $***P < 0.001$ (highly significant).

B. Supplementary tables

Table S1. Main elements of Cu-LDH measured by ICP-OES.

Cu	Mg	Al
0.122	0.257	0.061
μg mL^{-1}	μg mL^{-1}	μg mL^{-1}

Table S2. Zeta potentials of Cu-LDH, PEG-Cu-LDH, and PEG-Cu-LDH/HMME@Lips.

Cu-LDH	PEG-Cu-LDH	PEG-Cu-LDH/HMME@Lips
50.53	-3.031	-12.54

C. Supplementary figures

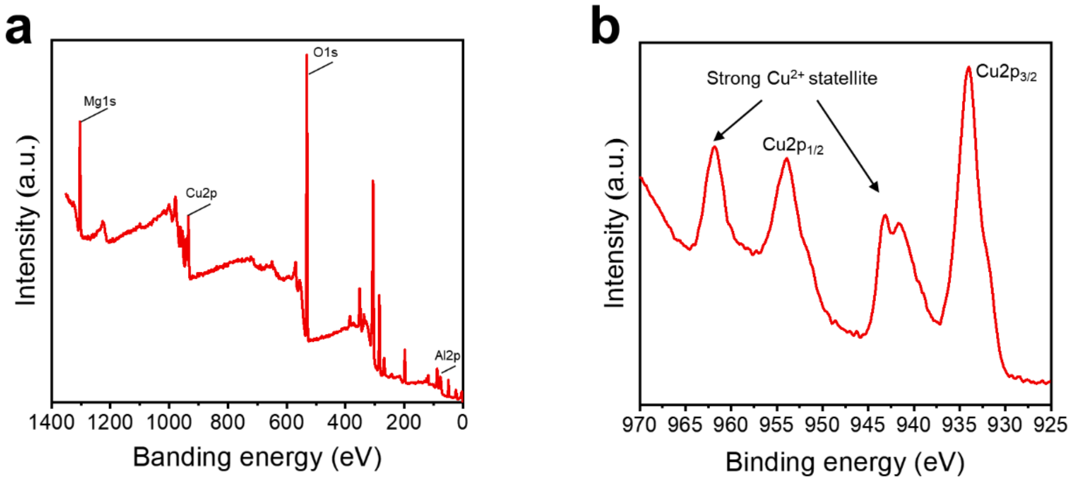

Figure S1. XPS spectra of Cu-LDH nanosheets: (a) survey, (b) Cu2p.

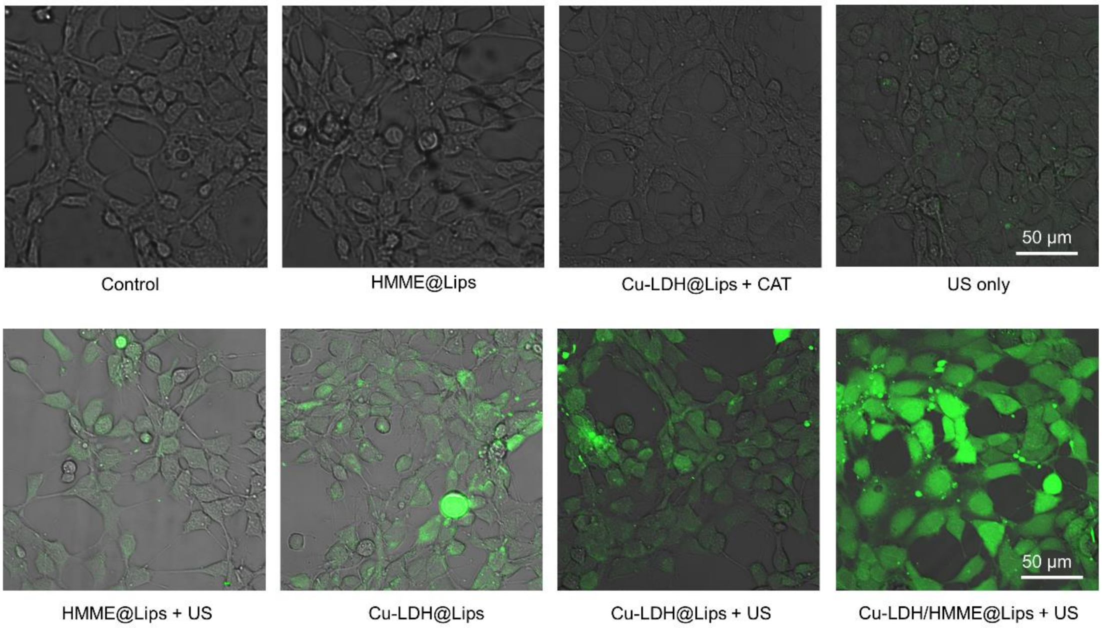

Figure S2. CLSM images of 4T1 cells stained with DCFH-DA after treatments under different conditions indicated.

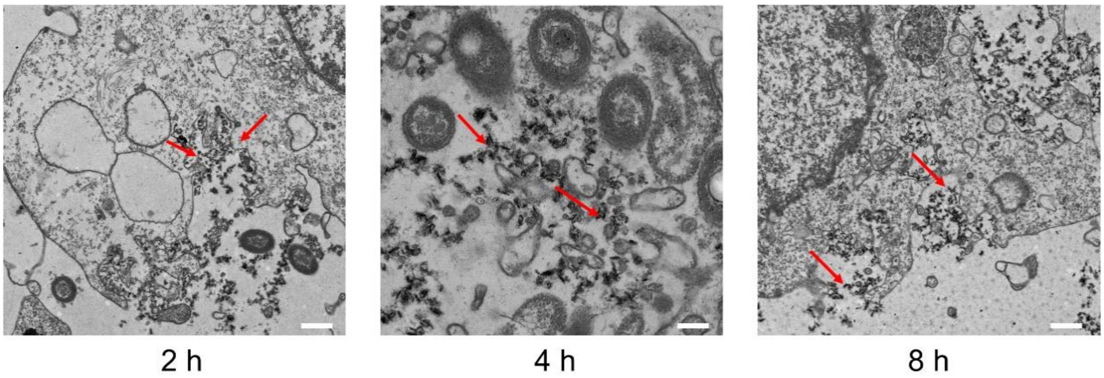

Figure S3. Bio-TEM images of 4T1 cells after coincubation with Cu-LDH@Lips for varied durations to observe internalization and excretion dynamics of them.

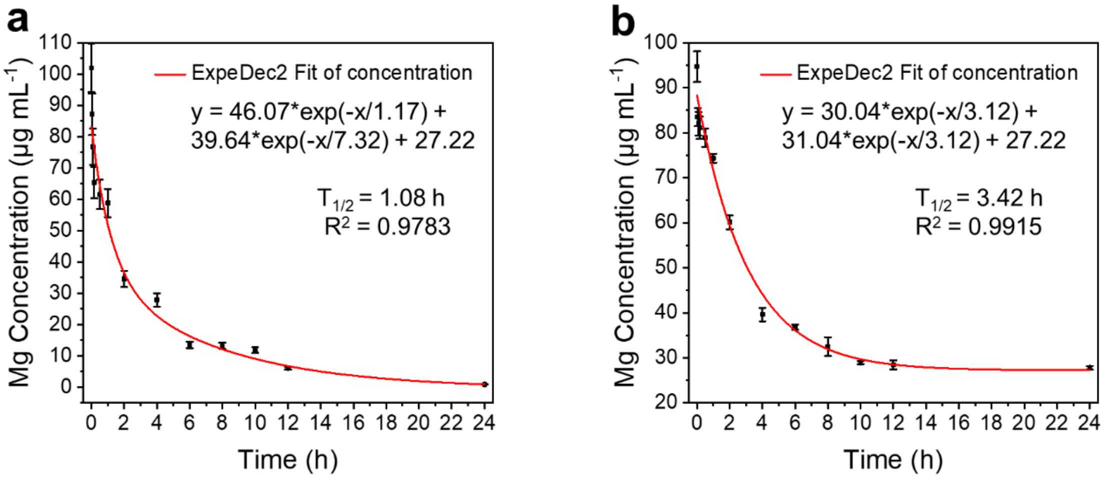

Figure S4. Blood-circulation lifetimes of (a) fresh Cu-LDH and (b) Cu-LDH@Lips after intravenous administration into mice (n = 3).

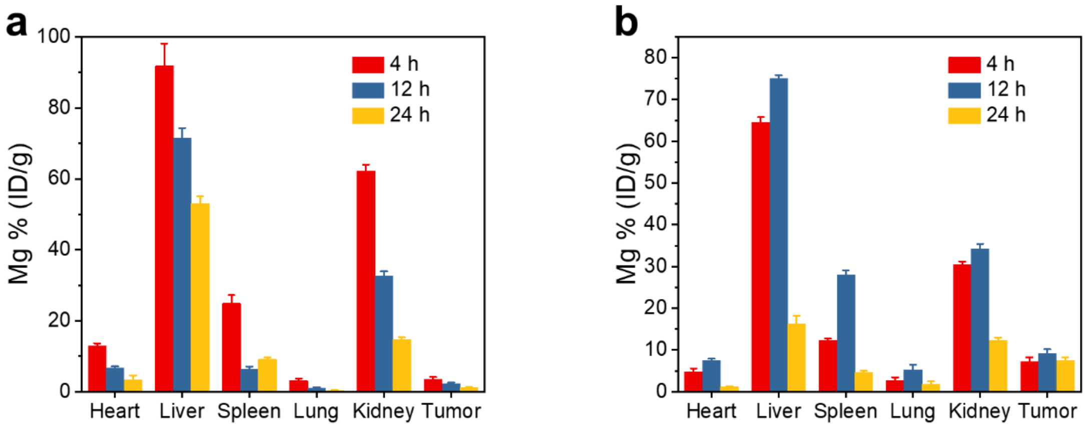

Figure S5. Biodistribiutions of Cu^{2+} after injecting (a) PEG-Cu-LDH@Lips (20 mg kg^{-1}) and (b) Cu-LDH@Lips (20 mg kg^{-1}) for 4, 8, and 24 h.

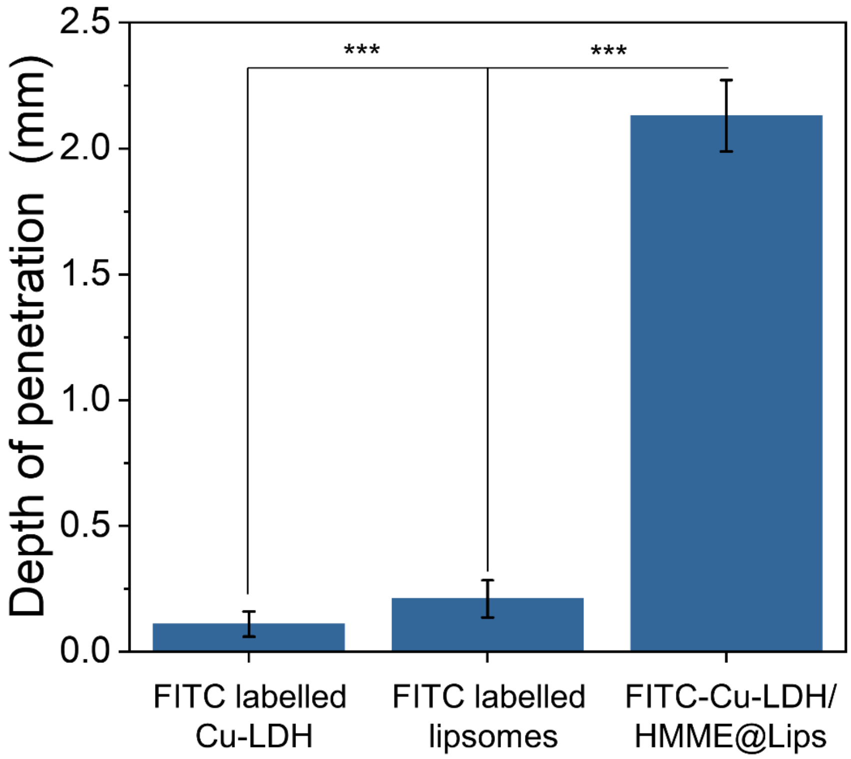

Figure S6. Data analysis of the average depth of nanoparticles infiltrating into the tumor based on the distribution of FITC green fluorescence in the tumor.

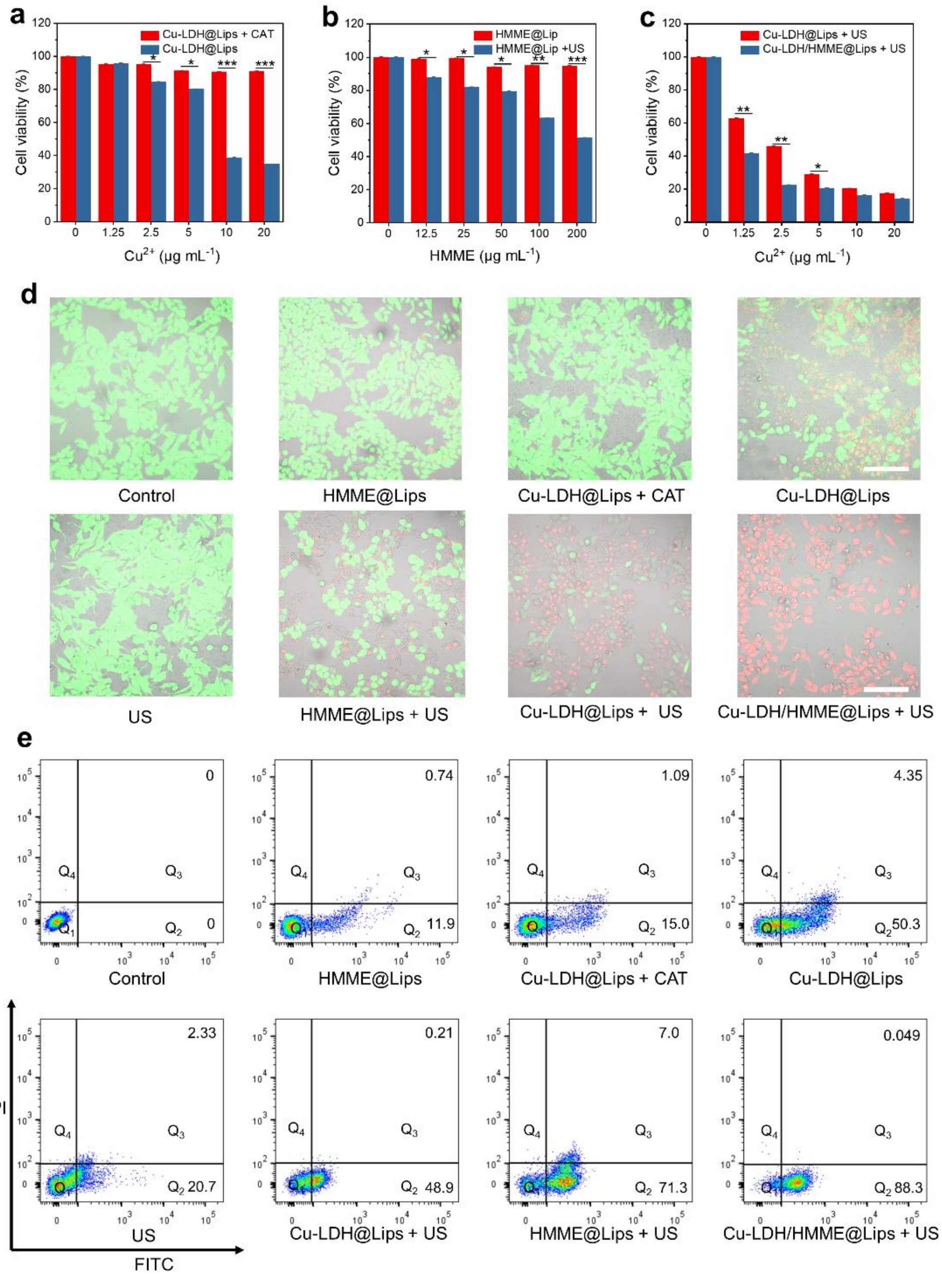

Figure S7. (a-c) *In vitro* cell viability of 4T1 cells treated with Cu-LDH@Lips + CAT and Cu-LDH@Lips at varied equivalent Cu^{2+} concentrations (a) with HMME@Lips and HMME@Lips + US at varied HMME concentrations (b) and with Cu-LDH@Lips + US or Cu-LDH/HMME@Lips + US at varied equivalent Cu^{2+} concentrations (c), for 24 h. (d) CLSM images of 4T cells stained by Calcine AM a PI after different treatments. Scale bar: 100 μm. (e) Flow cytometry analyses of the 4T1 cell apoptosis after various treatments.

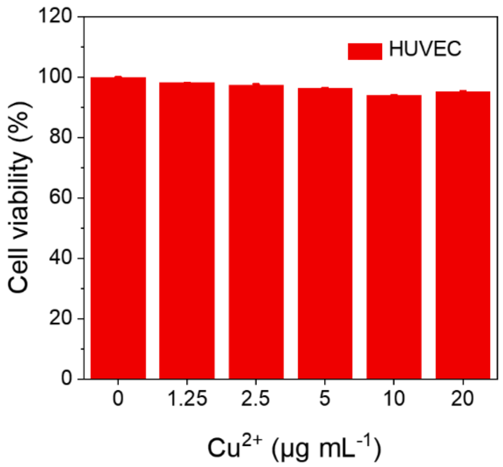

Figure S8. Cell viability of HUVEC cells after incubation with Cu-LDH/HMME@Lips at varied concentrations.

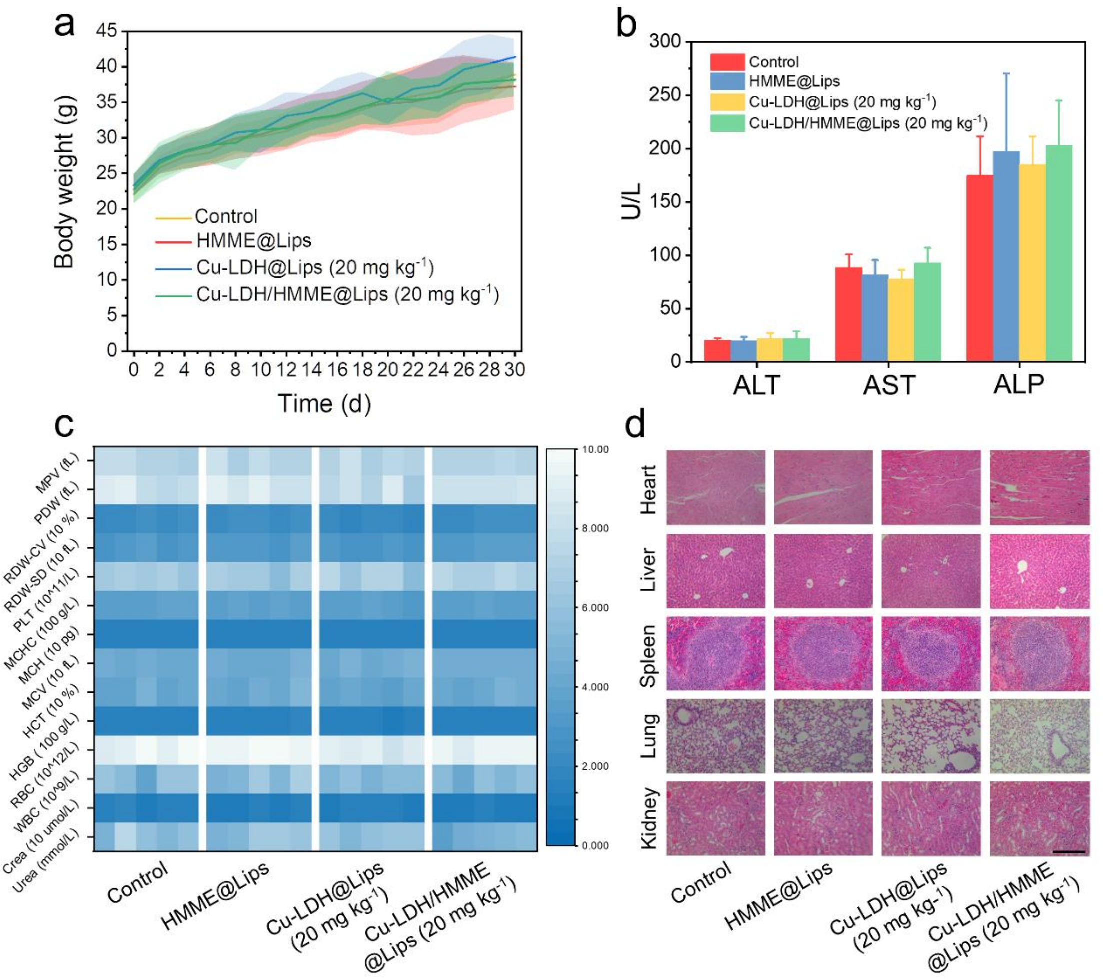

Figure S9. (a) Body weights of Kunming mice treated in different conditions during one-month feeding. (b, c) Hematological assays of Kunming mice after 30 days post-treatment with different agents. (d) Histological sections of main organs (heart, liver, spleen, lung, and kidney) obtained from the Kunming mice sacrificed on day 30 in varied post-injection groups, scale bar 200 μm.

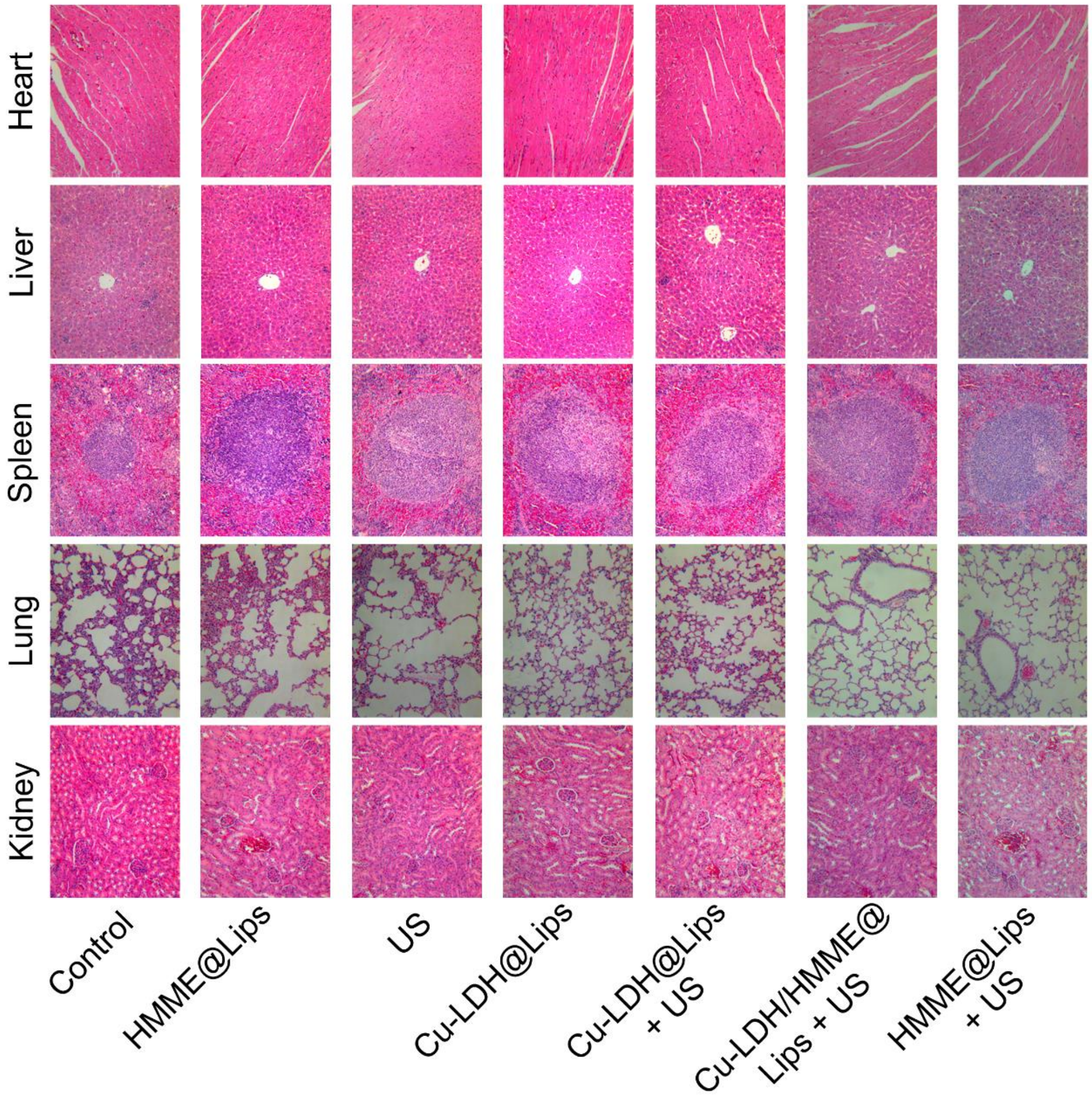

Figure S10. Histological sections (H&E stained) of main organs obtained from subcutaneous 4T1 tumor-bearing BALB/c mice, scale bar 200 μm.

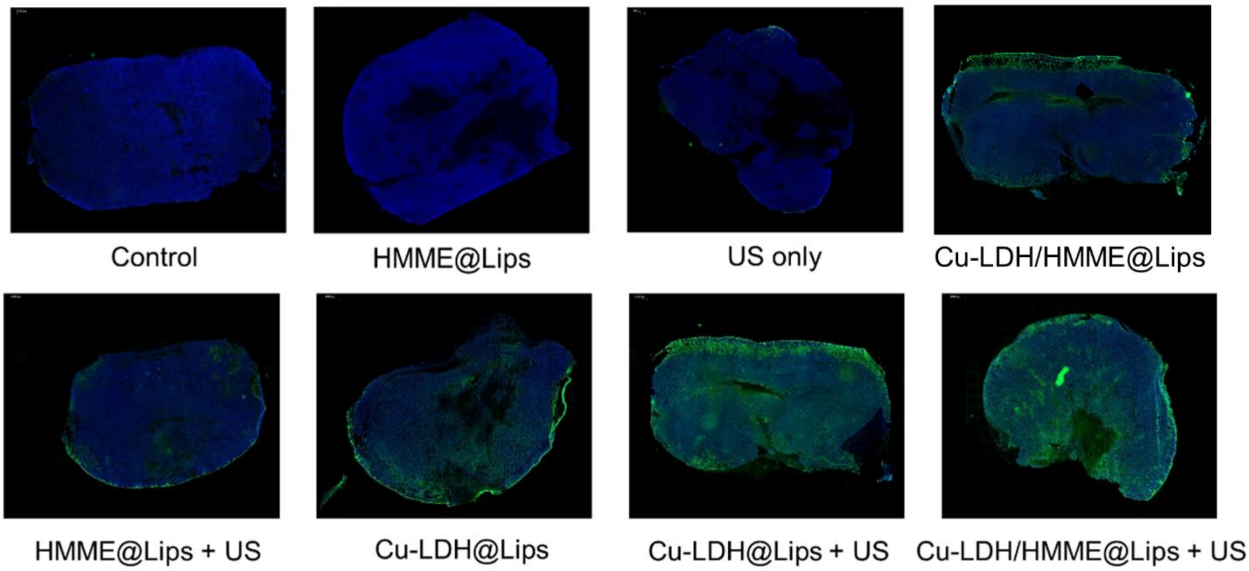

Figure S11. ROS immunofluorescence of tumor slices which were harvested from mice treated under different conditions.
